# Functionality of Immunoglobulin G and Immunoglobulin M Antibody Physisorbed on Cellulosic Films

**DOI:** 10.3389/fbioe.2017.00041

**Published:** 2017-07-17

**Authors:** Ziwei Huang, Vikram Singh Raghuwanshi, Gil Garnier

**Affiliations:** ^1^Department of Chemical Engineering, Bioresource Processing Institute of Australia (BioPRIA), Monash University, Clayton, VIC, Australia

**Keywords:** antibody, immunoglobulin G, immunoglobulin M, cellulose, physisorption, functionality, reflectivity, aging

## Abstract

The functionality and aging mechanism of antibodies physisorbed onto cellulosic films was investigated. Blood grouping antibodies immunoglobulin G (IgG) and immunoglobulin M (IgM) were adsorbed onto smooth cellulose acetate (CAF) and regenerated cellulose (RCF) films. Cellulose films and adsorbed IgG layers were characterized at the air and liquid interface by X-ray and neutron reflectivity (NR), respectively. Cellulose film 208 Å thick (in air) swell to 386 Å once equilibrated in water. IgG adsorbs from solution onto cellulose as a partial layer 62 Å thick. IgG and IgM antibodies were adsorbed onto cellulose and cellulose acetate films, air dried, and aged at room temperature for periods up to 20 days. Antibody functionality and surface hydrophobicity were measured everyday with the size of red blood cell (RBC) agglutinates (using RBC specific to IgG/IgM) and the water droplet contact angle, respectively. The functionality of the aged IgG/IgM decreases faster if physisorbed on cellulose than on cellulose acetate and correlates to surface hydrophobicity. IgG physisorbed on RCF or CAF age better and remain functional longer than physisorbed IgM. We found a correlation between antibody stability and hydrogen bond formation ability of the system, evaluated from antibody carbonyl concentration and cellulosic surface hydroxyl concentration. Antibody physisorbs on cellulose by weak dipole forces and hydrogen bonds. Strong hydrogen bonding contributes to the physisorption of antibody on cellulose into a non-functional configuration in which the molecule relaxes by rotation of hydophobic groups toward the air interface.

## Table of Contents Graphic


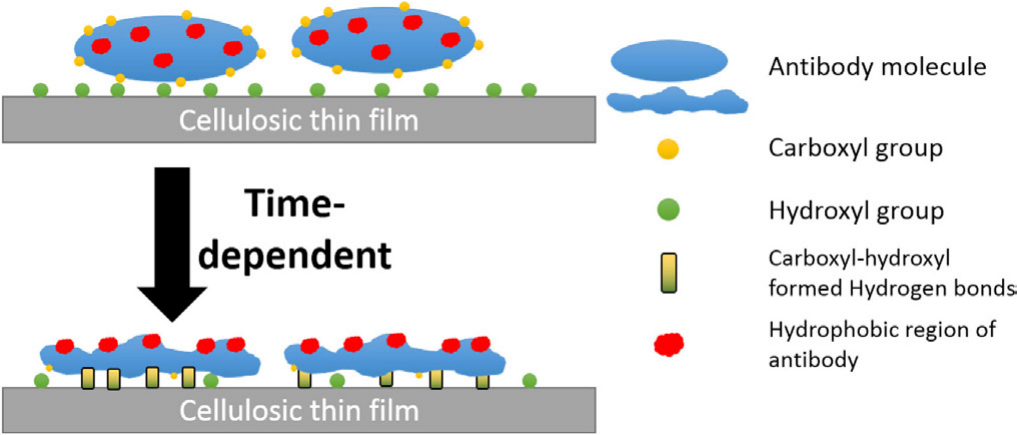


## Introduction

Paper is a remarkable platform to engineer a new generation of low cost and performant biomedical diagnostics for common, everyday analyses such as determining blood glucose, blood groups, and pathogens (Pohanka et al., 2007; Ali et al., 2009; Pelton, 2009; Then and Garnier, 2013a,b). Paper diagnostics typically involve the presence of physisorbed antibodies, enzymes, or functional biomolecules on paper. While enzymes on paper were shown to be remarkably robust (Khan et al., 2010a,b), antibody molecules such as blood typing immunoglobulin G (IgG) and immunoglobulin M (IgM) are much less stable once air-dried on paper (Guan et al., 2014). The adsorption, desorption, and longevity of functional biomolecules—especially antibodies—on paper are important phenomena to understand for engineering efficient diagnostics. Commercialization of paper diagnostics requires a shelf-life of around 1 year, while currently antibody physisorbed and dried on paper last about 1 month if stored under ambient conditions (Delaney et al., 2011; Huang et al., 2017). This protein functionality loss on paper under ambient conditions has not been well characterized and the aging mechanisms remain poorly understood. These issues are currently limiting the commercialization of the paper bio-diagnostics.

Paper is a 3-dimensional non-woven material made of macro, micro, and nanocellulose fibers (Ververis et al., 2007) bonded together mostly by hydrogen bonds. The void structure between the wettable cellulose fibers provides paper its unique capillarity benefiting microfluidics and diagnostics (Then and Garnier, 2013a,b). The type of lignocelluloses fibers and the uniformity of their distribution (formation), paper density, polymeric additives, and surface treatments dictate the paper chemical and physical properties (Klemm et al., 2004). While direct optimization of paper is a logical approach to enhance the performance of paper-based sensors and bio-devices, the paper structure obscures the detection of biomolecules such as antibody. Visualizing antibody morphology and quantifying its functionality on paper is difficult for many reasons. First, antibodies as macro-proteins, share an elemental polymeric structure not too dissimilar from cellulose (Cooper, 2000). Second, the critical dimensions of the antibody molecules, which range from 2 to 30 nm, are at least 7 orders of magnitude smaller than any dimension of cellulose fibers (length = 0.8–3 mm, diameter = 0.8–30 µm, roughness = 1–2 µm), and 3–4 orders smaller than paper pore size (1–100 μm) (Snell et al., 2001). Thus, with most analytical techniques, antibodies become insignificant from the paper or pulp fibers onto which they are adsorbed. Using smooth cellulosic films as model substrates is a way to alleviate the effect of paper morphology from the study (Su et al., 2015). Therefore, the variables in antibody functionality loss on nanoscale cellulosic films can be limited to chemical property and interactions.

Antibodies are functional proteins with four hierarchical levels of structure (Buxbaum, 2007). The functionality of an antibody is closely related with the localization of its active sites, which have a 3-dimensional structure fitting in complementary fashion the antigenic determinant (Singer and Doolittle, 1966; Tanford, 1968). Functionality loss represents some disruptions of hydrogen bonds, salt bridges, di-sulfide bonds, or non-polar hydrophobic interactions, which are the main bonds determining the quaternary, tertiary, and secondary structures (Ophardt, 2003). The partial secondary structure change is commonly observed in the antibody functionality loss induced by pH, salt, and temperature (Bai et al., 1998; Vermeer and Norde, 2000). The primary structure can only be affected by chemical reactions (Kubo, 1995). In blood-grouping paper diagnostics, antibodies are released from the substrate to trigger the agglutination within antigen-positive red blood cells (RBCs) (Jarujamrus et al., 2012). However, the partial release of antibodies was reported after long aging periods, leading to sensor invalidity (Sajid et al., 2015). This suggests an irreversible antibody adsorption on paper, affecting the antibody-antigen reactions.

Many analytical techniques can be used to quantify the adsorption of biomolecules on cellulose surfaces. These include quartz crystal microbalance, surface plasmon resonance, ellipsometry, grazing incidence X-ray diffraction, atomic force microscopy, X-ray (XR), and neutron reflectivity (NR) (Su et al., 2015). Among those methods, reflectivity using neutrons is a low-energy non-destructive characterization method of choice to visualize and quantify biomolecules at the solid/liquid and solid/air interface. It, however, requires a thin (5–100 nm) and smooth film (roughness of 1 nm). Neutrons are sensitive to the scattering cross section of hydrogen (H) and deuterium (D), allowing selective contrast of the interphase.

It is the primary objective of this study to quantify the effect of surface chemical composition and aging time on the functionality of the two main categories of blood typing antibodies (IgG and IgM) physisorbed onto cellulosic surfaces. Secondary objectives are to elucidate the mechanisms involved and to measure the thickness and morphology of the antibodies adsorbed directly onto cellulose. Thin and very smooth films of cellulose acetate (CAF) and regenerated cellulose (RCF) were used as model surfaces to explore the mechanism of functionality loss for physisorbed IgG and IgM. NR measurements were performed to determine the thickness and uniformity of the films and to quantify the layer thickness of adsorbed antibodies. Two novel methods were developed to monitor antibody functionality on the films and the surface hydrophobicity. The first one consists of quantifying the blood typing efficiency of antibodies by measuring the relative size of the agglutinates in antigen-positive RBCs. The second method developed entails recording the contact angle formed by water droplets over antibody films to relate surface hydrophobicity with aging time. Last, the carboxyl content of IgG and IgM was measured using pH titration to quantify hydrogen bonding interaction ability. Here, we aim at better understanding the mechanisms behind the aging of antibody physisorbed on cellulose to engineer paper more sensitive, cheaper, and more robust bio-diagnostics.

## Materials and Methods

### Materials

Cellulose acetate and acetone were purchased from Sigma-Aldrich. Polished silicon blocks of diameter 50.8 mm, thickness 12 mm, n-type Si (100) were acquired from SIL’TRONIX (Archamps Technopole, Archamps, France). Anti-A FFMU (for further manufacturing use, IgM antibody), anti-D FFMU (IgG antibody), and antihuman FFMU IgG (IgG antibody) were bought from Alba Bioscience (United Kingdom). For testing antibody blood grouping efficiency, CSL Revercell™ 15% (for IgM antibody) and Abtectcell™ III 3% (for IgG antibody) were purchased from bioCSL Pty Ltd. (Australia). Pierce™ 660 nm Protein Assay Reagent came from Thermo Scientific™, IL, USA.

### Cellulosic Thin Films

Cellulose acetate (0.5 g) was first dissolved fully in acetone (100 mL). This solution was used to spin coat films on the cleaned silicon wafers. Spin coating was performed on a WS-650-23B spin coater (Laurell Technologies Co., North Wales, PA, USA) setup at 4,000 rpm for 30 s. Afterward, the cellulose acetate coated Si block was kept overnight at 23°C in a sodium methoxide solution (1:50 dilution in methanol) to regenerate cellulose from the cellulose acetate (Su et al., 2015).

### Reflectivity Measurements

X-ray reflectometer measurements were recorded at the solid/air interface on a Panalytical X’Pert Pro instrument with Cu Kα X-ray source (λ = 1.54 Å). NR measurements were made on the PLATYPUS instrument of the Australian Nuclear Science and Technology Organization (ANSTO), Sydney, NSW, Australia. In a typical NR experiment, the Si/cellulose sample was mounted onto the cell filled with D_2_O. Data were collected at three angles 0.5°, 0.85°, and 3.8°. Data reduction and fitting of the NR curves were achieved using IgorPro based software MOTOFIT (Nelson, 2006). Full procedure is described in the previously published work (Su et al., 2016).

### Blood Typing Efficiency Measurement

Glass slides were plasma treated prior to spin-coating with the cellulose acetate solution to create a thin film (CAF) and regenerated into a cellulose film (RCF) following the same methodology. Anti-A FFMU and antihuman IgG FFMU were diluted into 1:4 solution with MilliQ water, which were spread over cellulosic films coated glass slides (CAF and RCF). All samples were air-dried and stored for aging in a controlled environment (23°C and 50% relative humidity) until tested. To test, on each day, a 3 µL droplet of CSL Revercell™ (A1 cells and B cells) was deposited onto the IgM-treated cellulosic surfaces. A coverslip was applied after mixing with the pipette tip for 10 s, and the agglutinates were observed by optical microscope (Olympus BX60). For cellulosic film treated with antihuman IgG FFMU antibodies, 15% Abtectcell™ III cells were incubated with Anti-D FFMU (1:20 dilution in MilliQ water) to sensitize cells (Yeow et al., 2015). Then, a 3 µL droplet of this cell solution was deposited onto the RCF- or CAF-coated glass slides. A coverslip was applied after mixing with the pipette tip for 10 s, and the agglutinates were observed by optical microscope. The antibody blood grouping efficiency was characterized by the size of the agglutinates with the antigen-positive cells, measured using the particle size function in ImageJ. Functionality is measured with the relative size of the sensitized RBC defined as Eq. 1.

(1) $$\text{Antibody functionality}=\frac{\text{Average size at time T}}{\text{Average size at time Day 0}}$$

### Surface Hydrophobicity

Surface hydrophobicity of both IgG and IgM treated surfaces was characterized by contact angle measured using a Contact Angle System (OCA 35, Dataphysics, Germany). Basically, a 3 µL droplet of distilled/deionized water was deposited on the surface and the equilibrium contact angle was measured after 10 s. The average of five measurements is reported.

### Antibody Characterization

The concentration of Anti-A FFMU and antihuman IgG FFMU was quantified using a Pierce™ 660 nm Protein Assay following the manufacturer’s protocol using BSA as model protein. The absorbance (Abs) at 660 nm was measured with a Cary 60 UV–Vis Spectrophotometer (Agilent Technologies, CA, USA). The carboxyl content was measured using pH titration (Excellence Titrator T5, Mettler Toledo). Anti-A FFMU and antihuman IgG FFMU were diluted to around 1 mg/mL. 0.1 g BSA was used as the model protein to validate the method. The pH of 40 mL solution was adjusted to around 4 with 0.1 M HCl, then titrated with 0.1 M NaOH to pH 11.5. The number of moles of NaOH needed to change the pH from the lower plateau to the higher plateau was then calculated, reflecting the mole of carboxyl groups neutralized in the protein. For the BSA, the theoretical carboxyl content in 1 mol BSA was calculated as:

(2) $$\frac{100\text{mol}\left(\text{COOH}\right)}{66\text{,}000g}=1.52\times 10^{-3}\text{mol/g}$$

## Results

**Figure 1:**
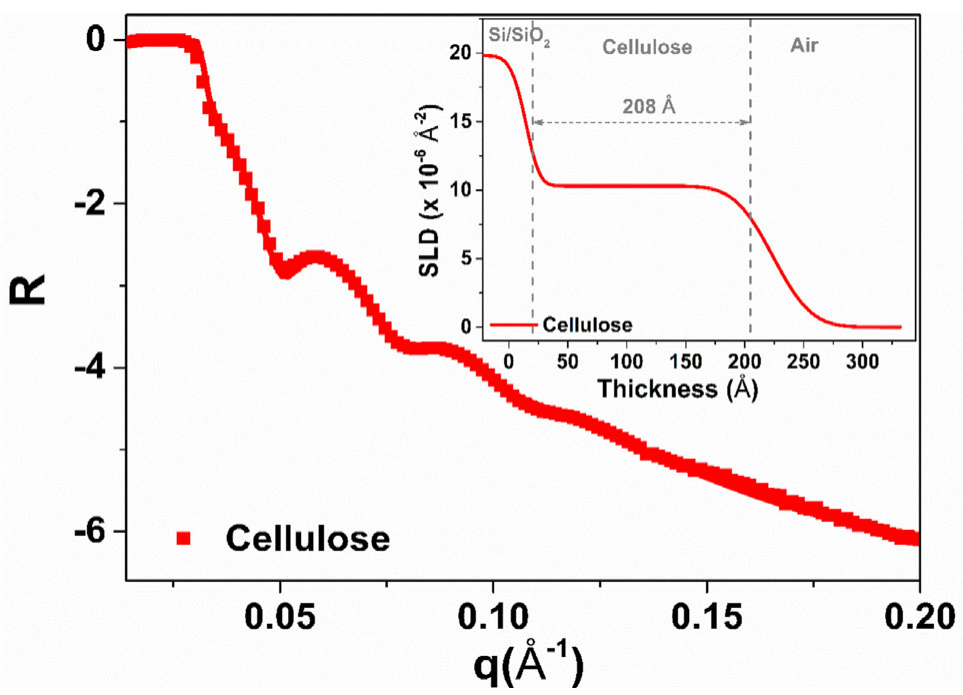
X-ray reflectivity of the regenerated cellulose thin film (air interface). The solid line shows the fit while the inset presents the scattering length density plot resulting from fitting the reflectivity curve.

The quality of the regenerated cellulose thin film spin-coated over the glass slides (RCF) was analyzed by X-ray reflectivity (XR). X-ray and NR are powerful methods to characterize thin films with respect to their thickness, roughness, scattering length density (SLD) variation, and volume fraction. Figure 1 shows the XR curve of RCF at the solid/air interface. The multiple fringes observed reveal that the film is homogeneous and smooth. Quantitative information is extracted by fitting the XR curve with a model using the Igor-based macro MOTOFIT (Nelson, 2006). A single layer model was used to fit the cellulose film layer with a preformed SiO_2_ layer on the Si block. The solid line in Figure 1 shows the fit and the figure inset highlights the respective SLD profile resulting from fitting. The film thickness is 208 ± 5 Å with a roughness of 22 ± 4 Å.

**Figure 2:**
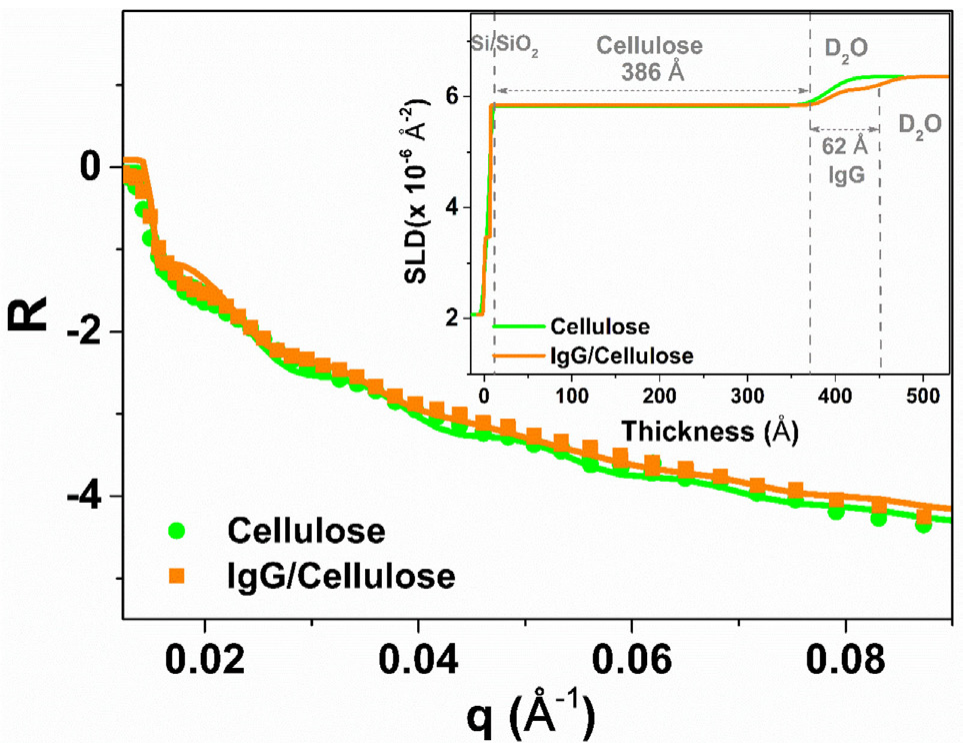
Neutron reflectivity curves of the regenerated cellulose film (RCF in green with filled circles) and with the adsorbed immunoglobulin G layer (orange, filled squares) on the RCF in D_2_O buffer. The solid line shows the fit and the inset represents the plot of scattering length density variation with respect to the layers thickness.

Neutron reflectivity measurement was performed to characterize the adsorption thickness and morphology of the IgG antibodies deposited onto the RCF surface at the solid/D_2_O interface. Figure 2 shows the NR curves for the RCF (green) and the adsorbed IgG layer (orange) on RCF. There is a small shift observed for the adsorbed IgG NR curve toward lower *q* values with respect to the RCF NR curve. This indicates the presence of IgG molecules adsorbed onto RCF. The NR scattering curves were also fitted with MOTOFIT macro. A sandwich structure was assumed, starting with a native SiO_2_ layer, followed by the RCF layer, the adsorbed IgG, and the D_2_O buffer. The NR curve of the cellulose (RCF) layer (green) was fitted with a single layer model. For the adsorbed IgG molecules, an additional layer was applied onto the RCF layer. The fitting variables were thickness, SLD, volume fraction, and roughness of the film. The solid line in Figure 2 shows the fitted NR curves with the inset representing the respective SLD profile as a function of thickness.

Table 1 compares the parameters from NR curve fitting. In the presence of D_2_O, the cellulose film swells up to 386 ± 10 Å, about twice its initial thickness. The SLD of the RCF of composition C_6_H_7_D_3_O_5_ is 3.67 × 10^−6^ Å^−2^, due to three exchangeable hydroxyl groups exchanging protons (H) for the D’s of D_2_O; this was first reported by Kent et al. (Cheng et al., 2011) and further demonstrated in our laboratory (Su et al., 2015). The biomolecules also have exchangeable amines able to interchange their mobile proton (H) for deuterium (D), which leads to an increase in their SLD. The IgG adsorbed layer is 62 ± 4 Å thick with a SLD of 4.17 × 10^−6^ Å^−2^ (Table 1). The SLD variation in the inset of Figure 2 shows a small difference between SLD of IgG and cellulose in D_2_O. Therefore, only a small change is observed in the respective NR curves.

**Table 1:** Cellulose film and adsorbed immunoglobulin G (IgG) antibodies layers characteristics as measured from NR curves fitting.

Film	Thickness (Å)	Scattering length density (10^−6^ Å^−2^)	Volume fraction	Roughness (Å)	χ^2^
Regenerated cellulose film (RCF)_air (XR)	208 ± 5	–	–	22 ± 4	0.002
RCF_D_2_O (NR)	386 ± 10	3.67	20 (cellulose)	15 ± 4	0.02
IgG_D_2_O (NR)	62 ± 5	4.17	15 (IgG)	10 ± 4	0.01

The effect of surface composition and aging on the functionality of IgG antibodies adsorbed onto cellulosic surfaces was quantified using two novel techniques. We discovered two interesting phenomena while experimenting antibodies physisorbed onto various surfaces. The first phenomena is that layers of dry antibodies become hydrophobic as they age. The second is that the size of the aggregates formed within antigen-positive RBCs decreases as the antibody ages and loses its functionality. These two observations were developed into simple, yet sensitive and reproducible techniques to quantify the effect of antibody aging on antibody functionality and selectivity.

**Figure 3:**
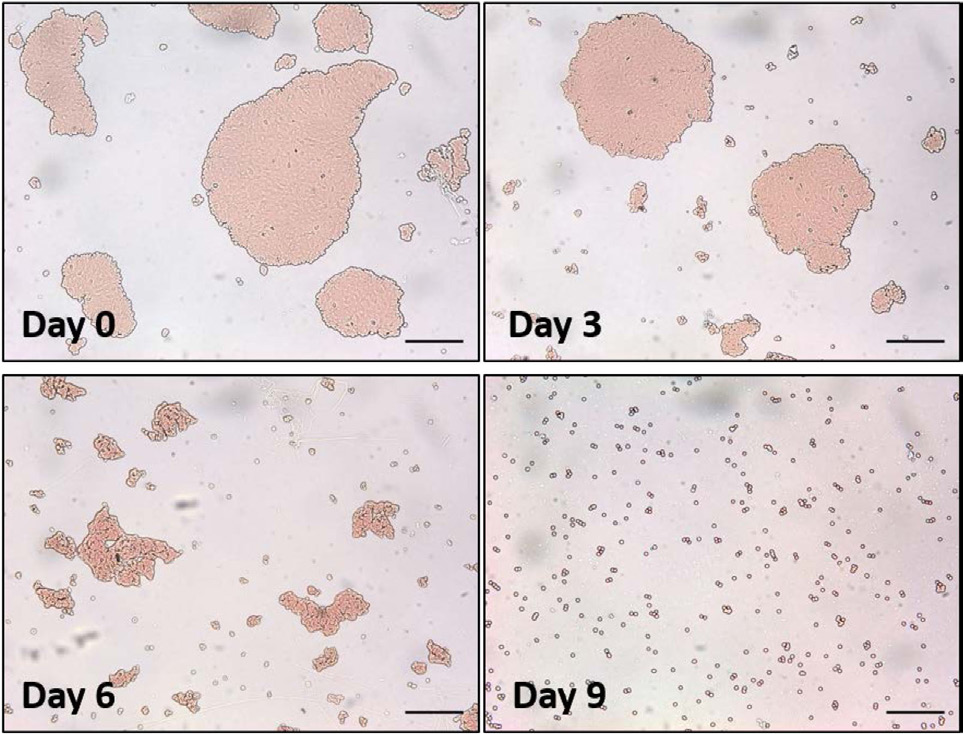
Optical microscopic images of a droplet of sensitized red blood cells (D-RBCs) over antihuman immunoglobulin G coated regenerated cellulose film with respect to aging time. The bar on each micrograph represents a distance of 100 µm.

In our first method, a droplet of antihuman IgG antibodies was deposited onto the surface of smooth RCF and cellulose acetate film (CAF) and dried. The protein concentration of the IgG solution was 2.75 mg/mL. These films were aged in the controlled environment for up to 1 month. To measure antibody activity after a given aging period, a fresh droplet of sensitized red blood cells (D-RBCs) was deposited onto the surface of the aged IgG-treated cellulosic film and allowed to agglutinate. The size of the agglutinates formed within D-RBCs was observed to change over time (Figure 3).

**Figure 4:**
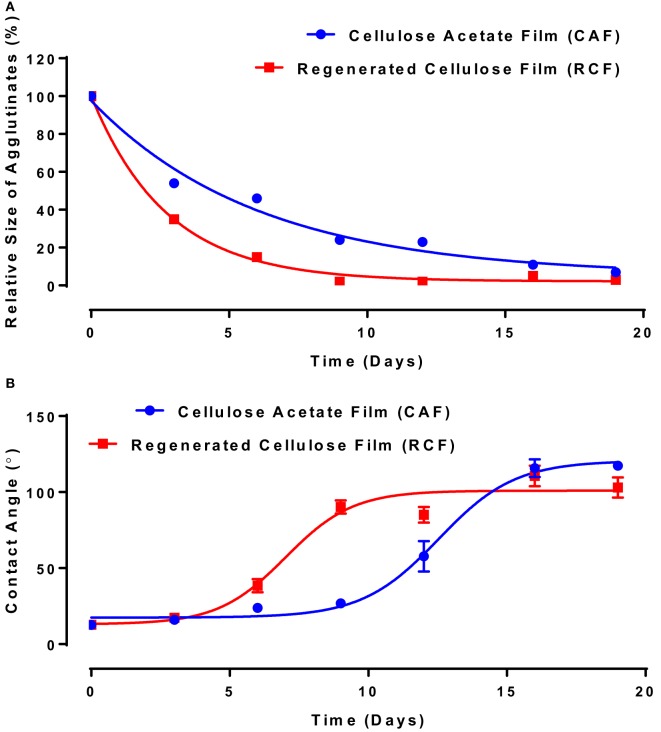
Effect of aging time and surface properties on the hydrophobicity and functionality of immunoglobulin G (IgG) antibody adsorbed on cellulosic films. Red, cellulose acetate film (CAF); blue, regenerated cellulose film (RCF). **(A)** Effect of IgG antibody aging on the relative size of agglutinates on CAF and RCF surfaces. Data were fitted with a one-phase decay, with *R*^2^ of 0.9778 (CAF) and 0.9965 (RCF). **(B)** Effect of IgG antibody aging on surface hydrophobicity on CAF and RCF. Data were fitted with Boltzmann function, and the *R*^2^ are 0.988 (CAF) and 0.937 (RCF).

Image analysis was used to measure the average size of agglutinates, which were compared to the average original size of blood agglutination over the fresh IgG layer surface. Since the average blood agglutinate size decreases as IgG ages and loses its ability to bind antigen-positive cells, antibody functionality is represented as the relative size of agglutinates formed with D-RBCs (Eq. 1). The functionality of IgG antibody decreases on both CAF and RCF with respect to the number of aging days as shown in Figure 4A. On both CAF and RCF, the relative size of agglutinates decreases from the original value of 100% to almost 0% within 19 days of aging. For the second method, the wettability of the antibody-coated model surfaces was measured at different aging periods. Droplets of water were deposited onto the two surfaces and the equilibrium contact angle was recorded. The surface hydrophobicity presented as the contact angle formed by a water droplet on the IgG treated surfaces also changes within the 19 days of aging (Figure 4B). The hydrophobicity of RCF samples increases with aging to reach a plateau on day 9 and the plateau of CAF is reached on day 16.

**Figure 5:**
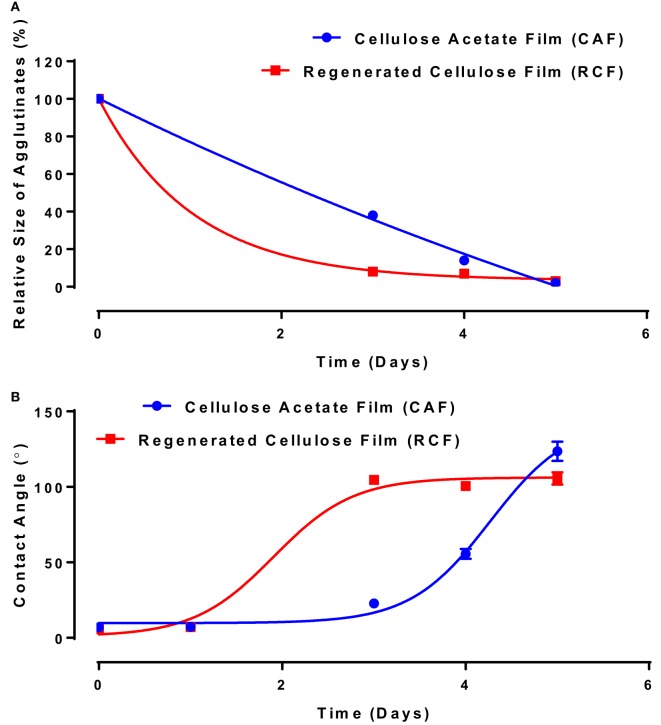
Effect of aging time and surface properties on the hydrophobicity and functionality of immunoglobulin M (IgM) antibody adsorbed on cellulosic films. Red, cellulose acetate film (CAF); blue, regenerated cellulose film (RCF). **(A)** Effect of IgM antibody aging on the relative size of agglutinates on CAF and RCF. Data were fitted with a one-phase decay model, with *R*^2^ of 0.9967 (CAF) and 0.9993 (RCF). **(B)** Surface hydrophobicity (contact angle) with respect to aging time. Data were fitted with Boltzmann function, with *R*^2^ of 0.999 (CAF) and 0.996 (RCF).

In the second study, the functionality of anti-A IgM antibody solution (3.07 mg/mL) physisorbed on RCF and CAF surfaces was studied using the same methodology. IgM is known in the industry to be much more potent antibodies for blood typing than IgG. The relative size of the blood agglutinates with respect to the aging time—representing antibody functionality change—is shown in Figure 5A. With antibody aging, the relative size of RBC agglutinates decreases on both RCF and CAF surfaces. On RCF, the relative size of agglutinates reaches almost 0 on day 3, while it lasts up to 5 days on CAF. The water contact angle representing the change in surface hydrophobicity is shown in Figure 5B. The antibody hydrophobicity increases non-linearly on both CAF and RCF surfaces as a function of aging time. The highest contact angle was observed on day 3 for RCF, and on day 5 for CAF.

**Figure 6:**
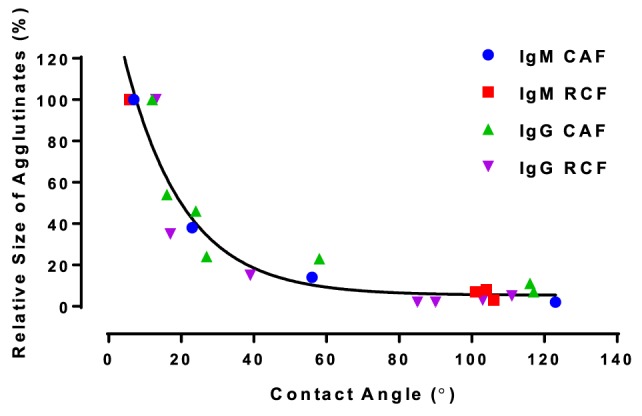
Functionality of immunoglobulin G (IgG) and immunoglobulin M (IgM) antibodies adsorbed on model surfaces (measured as relative size) as a function of surface hydrophobicity (contact angle) for regenerated cellulose film (RCF) and cellulose acetate film (CAF) surfaces. All data fits a one-phase decay master curve, $Y=5.355+150.312e^{-\frac{x}{16.383}}$, with *R*^2^ = 0.904.

Analysis of Figures 4 and 5 suggests a commonality between antibody hydrophobicity and functionality. Here, we raise the hypothesis that antibody functionality is related to surface hydrophobicity and that IgG and IgM antibodies both age by the same mechanism. All data from Figures 4 and 5, combining IgG and IgM aged over CAF and RCF, were, therefore, re-plotted as the relative size of the blood agglutinates vs. the water droplet contact angle on two surfaces (Figure 6). A master curve appears, which can be fitted by a simple one-phase decay model (*R*^2^ = 0.904). This supports the hypothesis of a common degradation mechanism for both IgG and IgM.

**Figure 7:**
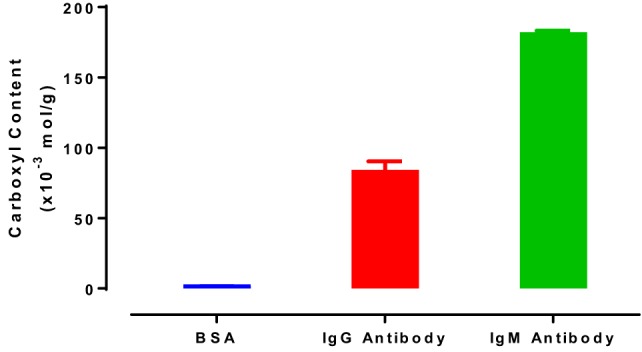
Carboxyl content of bovine serum albumin, immunoglobulin G (IgG) antibody (antihuman IgG, FFMU), and immunoglobulin M (IgM) antibody (anti-A FFMU), measured by pH titration.

The carboxyl groups of proteins were previously reported as participating to the interaction between protein and hydrophilic surfaces (Song et al., 2012). The carboxyl contents of IgG and IgM antibodies were then measured by pH titration to quantify the effect of antibody carboxyl content on the interaction with RCF and CAF surfaces (Figure 7). Bovine serum albumin (BSA) was also analyzed as reference protein (Figure 7). A BSA molecule contains 100 free carboxyl groups, referred to as 100 mole carboxyl groups per mole of BSA (Saroff et al., 1953). As the BSA molecular weight is 66 kDa (Sigma Alrich), the carboxyl content would be 1.52 × 10^−3^ mol/g (mole carboxyl groups per gram of protein, Eq. 2). This value is similar to the result displayed in Figure 7 (1.58 ± 0.07 × 10^−3^ mol/g). Results are presented as the number of moles of carboxyl groups per gram of protein. IgG antibody contains 84.24 ± 6.19 × 10^−3^ mol/g, while the IgM antibody has the highest carboxyl content (182.19 ± 1.12 × 10^−3^ mol/g).

## Discussion

Thin films of cellulose acetate (CAF) and regenerated cellulose (RCF, from cellulose acetate) were spin-coated. The quality of the RCF films in terms of smoothness, thickness, and roughness was characterized by XR (Figure 1). Thin (208 ± 5 Å) and smooth (roughness of 16 ± 4 Å) cellulosic films were achieved as recorded by XR measurement at the cellulose/air interface. NR measurement at the film/D_2_O interface reveals the RCF film to swell in water and its thickness to increase up to 386 ± 10 Å, almost doubling. Swelling is due to the penetration of water within the cellulose chains relaxing and equilibrating in water from their unstable state under which they have been locked during the rapid spin coating/drying process. NR measurement upon IgG adsorption (Figure 2) from solution onto the RCF surface shows the formation of a partial layer of IgG as 62 ± 5 Å thick. Only a small difference between the NR curves of RCF with and without adsorbed layer of IgG was observed. This is due to the minor difference in SLDs of RCF (3.67) and IgG (4.17), providing small contrast between the two phases, combined with the thinness of the antibody layer. This contrast between cellulose surface and antibodies can be enhanced by deuterating either the antibodies or the cellulose by replacing H with D. Deuteration of cellulose was shown to significantly enhance SLD contrast between cellulose and protein, thus improving visualization and quantification of biomolecule adsorbed on cellulose; the methodology can be found elsewhere (Su et al., 2016; Raghuwanshi et al., 2017).

The functionality change of IgG and IgM antibodies was quantified during aging on two model surfaces (RCF and CAF) at constant temperature and humidity (23°C, 50% relative humidity). An IgM antibody molecule has a circular shape composed of a central circular region (190 ± 20 Å) connected to five sets of radial arms (110 ± 10 Å) (Czajkowsky and Shao, 2009). It has more than twice the bridging length of an IgG antibody (130 Å × 80 Å × 20 Å). Therefore, different physic-adsorption phenomenon of IgG and IgM can be expected on cellulosic films.

The functionality of both IgG and IgM lasts longer on CAF than on RCF surfaces (Figures 4 and 5). The difference between CAF and RCF suggests antibody oxidization to be an unlikely mechanism for the antibody functionality loss on films. Antibody oxidization can occur with proteins targeted by reactive oxygen species (ROS) (Lund et al., 2011). ROS can oxidize antibodies by forming carbonyl derivatives, sulfhydryl groups and crosslinking proteins (Lund et al., 2011). As the IgG and IgM samples were stored under identical conditions, the environment oxidization potential is identical for both antibodies and on both substrates. The significant difference in functionality observed between antibodies physisorbed on CAF and RCF rather suggests a dominant effect of the substrate ability to interact with the antibody over oxidation. A major difference between RCF and CAF lies in the surface content of hydroxyl groups. The regeneration of CAF into RCF results in the substitution of the 3 acetyl groups of cellulose acetate by 3 hydroxyl groups (Su et al., 2015). Hence, hydroxyl groups may be involved in the functionality loss of physisorbed antibody, which highlights the role of protein–surface hydrogen bond in the functionality loss mechanism. The master curve of antibody functionality versus surface hydrophobicity (Figure 6) also suggests that both IgG and IgM antibodies degrade by the same mechanism.

Rankl et al. (2006) reported antibodies to reversibly adsorb onto cellulosic films (trimethylsilyl ether cellulose, cinnamate-trimethylsilyl ether cellulose, and aminopropyl-trimethylsilylether cellulose) with a conformational change occurring upon air-drying, which exposes hydrophobic regions toward the hydrophobic air interface. Additionally, partial release of antibodies from a cellulosic substrate was reported in an aged sensor (Sajid et al., 2015). Hence, the declined antibody functionality represents a fraction of the proteins adsorb irreversibly to cellulosic surfaces. Here, we report this fraction increases in a time-dependent manner, with the rate affected both by the surface hydroxyl concentration and the antibody/protein carboxyl concentration. The conversion of reversible to irreversible adsorption depends on the increases in the amount of protein–surface hydrogen bonds. This time-dependent protein–surface interaction was also reported by Kurrat et al. (1997) for BSA adsorbed onto silica-titania. This transition in activity was found to correspond to an increase in hydrophobicity for antibody physisorbed on a surface.

**Figure 8:**
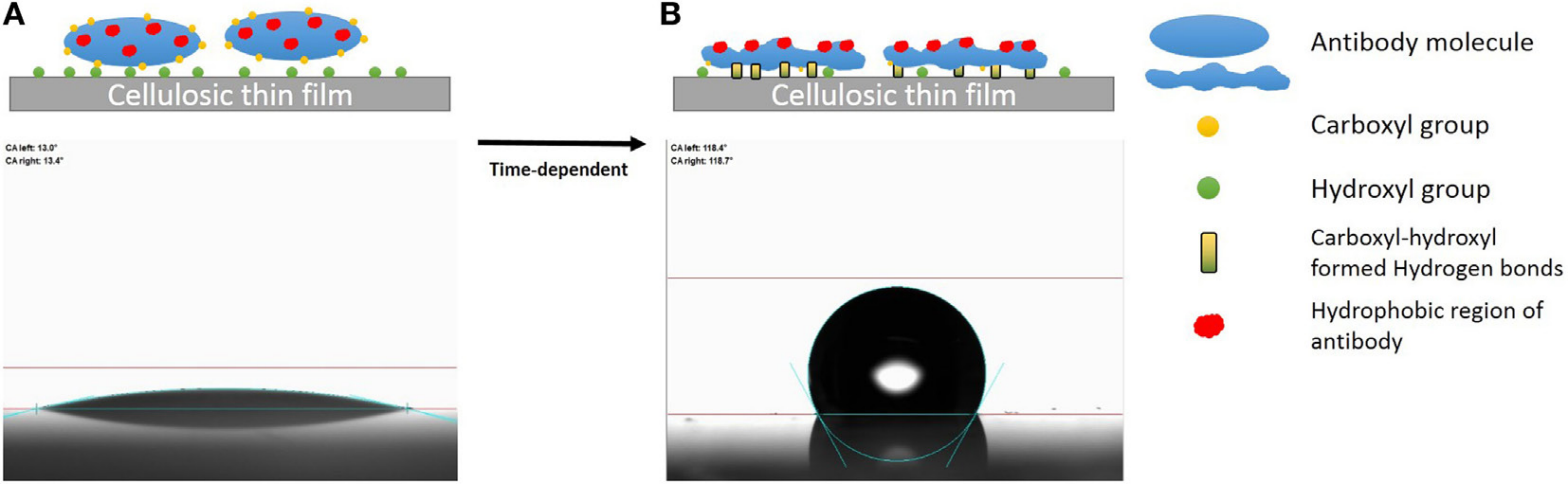
Schematic representation of the mechanism of antibody degradation on cellulose. **(A)** Antibody reversibly adsorbed onto a cellulose surface, which causes little change in the water contact angle as the fresh protein is hydrophilic. **(B)** Aging the antibody on cellulose has two effects. First, a time-dependent reconformation of the protein exposing its hydrophobic entities toward the protein/air interface occurs, thus increasing hydrophobicity as a function of time. Second, protein and cellulose develop irreversible adsorption through the stronger hydrogen bonds formed between the carboxyl groups of antibody and the hydroxyl group of the cellulosic surface.

Hydrogen bond between the carboxyl group of BSA and the surface hydroxyl groups or surface-bonded water molecules was reported by Song et al. (2012). The carboxyl groups from protein were stated to play an important role in adsorption, which was further reported by Penna et al. (2014) as initiating irreversible adsorption by computer modeling. The carboxyl content of IgM is indeed twice that of IgG (Figure 7), while IgM antibody functionality decreases much faster on both substrates than IgG antibody does (Figures 4A and 5A). This supports the role played by protein carboxyl content in the functionality loss of physisorbed antibody. Based on this analysis, our current understanding of the aging mechanism of physisorbed antibody is the following. Antibody, IgG or IgM, physisorb on cellulosic surfaces by weak dipole forces including hydrogen bonding. As aging progresses, two phenomena occur. The first is the establishment of stronger antibody-surface hydrogen bonds, which are proportional to the hydrogen bonding ability of the system. This hydrogen bonding ability of the system is related to the protein carboxyl concentration and the surface hydroxyl concentration. These H bonds define the antibody adsorption into a flatter and more rigid conformation than in solution. The second is a change in the physisorbed antibody conformation involving orientating some hydrophobic groups of the protein toward the air interface, and possibly protein unfolding. This is an effort of the system to minimize the free energy of its antibody–air interface with a higher entropic contribution from the protein re-conforming on the solid surface. These two conformation changes correspond to the drop in antibody functionality (ability to bind with RBC antigen) and the increase in surface hydrophobicity. This mechanism of antibody functionality loss is schematically represented in Figure 8.

## Conclusion

Thin films of cellulose acetate (CAF) and regenerated cellulose (RCF) were developed as model surfaces to investigate the adsorption behavior of IgG and IgM antibodies on cellulosic surfaces. Reflectivity (XR and NR) measurements reveals smooth films of roughness lower than the critical dimension of the antibody. These features are suitable to study antibody and biomolecule adsorption. Cellulose films 208 Å thick in air swells up to 386 Å once equilibrated in water. A partial IgG monolayer 62 Å thick was measured on cellulose films by neutron reflectometry. The functionality of both IgG and IgM antibodies adsorbed onto the CAF and RCF surface was determined by monitoring the blood typing efficiency of the antibodies with a novel method based on the relative size of the agglutinates formed by antigen-positive RBCs. The contact angle formed by water droplets deposited on these IgG and IgM antibody layers aged on the two cellulosic surfaces under ambient conditions was measured as a function of time (1–30 days). The functionality of both IgG and IgM antibodies decreases, while surface hydrophobicity increases with aging time. The master curve formed by the correlation between antibody functionality and surface hydrophobicity suggests that IgG and IgM antibodies interact with the cellulosic surface in the same manner and age by similar mechanisms. However, IgM shows a higher carboxyl content and a shorter stability on both cellulose acetate and cellulose surfaces than does IgG, suggesting the involvement of carboxyl groups in antibody functionality loss.

Our current mechanistic understanding is that antibody (IgG or IgM) physisorbs on cellulose by dipole interaction and hydrogen bonds. As aging progresses, two phenomena occur. First, the establishment of stronger antibody-surface hydrogen bonds proportional to the hydrogen bonding ability of the system. Second, the physisorbed antibody changes conformation by rotation of some hydrophobic groups toward the air interface and possibly protein unfolding. These two conformational changes correspond with the drop in antibody functionality and the increase in surface hydrophobicity with time. This study offers new methods of quantifying the functionality of antibody physisorbed on surfaces. It also provides a new perspective to optimize adsorbed antibody stability by preventing functionality loss through surface engineering.

## Author Contributions

ZH: made cellulose and cellulose acetate thin film samples using spin coating. Performed antibody functionality and surface hydrophobicity on film surface with respect to the size of RBC agglutinates (using RBC specific to IgG/IgM) and the water droplet contact angle measurements. ZH also invented the pH titration method for measuring carboxyl content of proteins. VR: performed X-ray and neutron reflectivity experiments and supervised ZH in preparation of thin films. GG: supervision of the work and discussion on mechanisms of antibody functionality and sorption phenomenon on cellulose and cellulose acetate film interface.

## Conflict of Interest Statement

The authors declare that the research was conducted in the absence of any commercial or financial relationships that could be construed as a potential conflict of interest.
